# Fear of COVID-19 among oral health professionals in South Africa

**DOI:** 10.4102/hsag.v29i0.2619

**Published:** 2024-05-31

**Authors:** Siphokazi M. Matomane, David P. Motloba

**Affiliations:** 1 Department of Community Dentistry, School of Oral Health Sciences, Sefako Makgatho Health Sciences University, Tshwane, South Africa

**Keywords:** COVID-19, pandemic, fear, oral health professionals, South Africa

## Abstract

**Background:**

The coronavirus disease 2019 (COVID-19) pandemic caused widespread psychological morbidity, especially among frontline workers. Oral health professionals (OHPs) are at increased risk of contracting and dying from COVID-19 because pf their proximity to infected patients. The objective of this study was to measure the level of COVID-19 fear and its predictors among South African OHPs.

**Aim:**

To evaluate the severity of fear of COVID-19 among OHPs in South Africa.

**Setting:**

Online platforms.

**Methods:**

In this cross-sectional study, a Google Forms-based online survey was conducted. The fear scale of COVID-19 (FCV-19S) was used to assess the level of fear among OHPs. Statistical data analysis was performed using SPSS 28.0. The multivariate logistic regression model was developed to assess the predictors of COVID-19 fear.

**Results:**

A total of 462 OHPs participated in this study. Approximately half of the participants, 212 (45.6%), indicated having severe fear of COVID-19. The regression model showed a significant association between COVID-19 fear and increased age, clinical experience, employment sector, professional group, positive COVID-19 test and COVID-19 vaccination.

**Conclusion:**

The results indicate that South African OHPs had low levels of corona phobia. Females, older and vaccinated OHPs had the greatest odds of COVID-19 fear.

**Contribution:**

This study provides insights into the severity of fear and anxiety experienced by OHPs in the time of the pandemic. The findings will aid in the development of appropriate interventions and programmes to deal with similar outbreaks in the future.

## Introduction

The coronavirus disease 2019 (COVID-19) pandemic epitomises one of the greatest health, social and economic catastrophes in recent times. The high COVID-19 contractibility, morbidity and mortality placed global health institutions under enormous pressure, with some organisations on the brink of collapse. Similarly, healthcare workers (HCWs) succumbed to a plethora of physical, emotional and psychological challenges because of COVID-19. These outcomes are attributed to long working hours, increased workload, perceived lack of safety and isolation from friends and family (Giusti et al. 2020; Si et al. 2020). Literature indicates that consequences of the prolonged exposure to these working conditions impacted the mental health of HCWs (Huang et al. 2021; Robles et al. 2020). Previous pandemics like Spanish flu, which presented in a similar manner to COVID-19, are associated with a high prevalence of anxiety, stress, depression and other forms of mental diseases (Leon & Marika 2021). It is well established that dentistry is a stressful profession because of a myriad of risks and stressors imminent in the settings (Agrawal et al. 2014). The threat of COVID-19 infections signifies a serious biological risk for oral health professionals (OHPs) (Banakar et al. 2020). During the initial waves of COVID-19, OHPs were obligated to provide urgent and emergency dental services (Meng, Hua & Bian 2020). In the absence of any effective treatment or vaccines at the time, COVID-19 morbidity and mortality were inevitable among professional groups. Prolonged contact with patients and the creation of aerosols, droplets and fomites from the use of ultrasonic scalers, handpieces and air-water syringes increase the risk of viral transmission in these settings (Aldahlawi & Afifi 2020). Despite standard preventative measures, the risk of COVID-19 is always present in dental settings. The timing of this study coincided with challenges around the availability and affordability of personal protective equipment (PPE), especially in private practice. The cost of PPE was exorbitantly high and not fully funded by medical schemes. According to Collin et al. (2021), this constitutes an institutional betrayal, as powerful institutions like medical schemes fail those who rely on them for safety. Treating patients during the pandemic placed additional health, financial and psychological burdens on OHPs. Global studies indicate that COVID-19 has had a serious psychological impact on health professionals (Giusti et al. 2020; Huang et al. 2021; Si et al. 2020). There is a dearth of evidence and limited data on the levels and state of COVID-19-related fear and anxiety among dentists globally. Combined studies of health professionals indicated that approximately 50% of dentists have experienced some form of stressful events, including psychological stress (Collin et al. 2021), occupational stress (Ge et al. 2020) and burnout (Özarslan & Caliskan 2021). The few recent studies among dentists indicate moderate to high levels of fear and anxiety related to COVID-19. According to Suryakumari et al. (2022), 58.3% of Indian dentists presented with mild corona-phobia as compared to Pakistani counterparts who had extreme fear and anxiety (Majeed et al. 2021). To date, limited research has been undertaken on the psychological impact of COVID-19 on OHPs in South Africa. The current study aims to assess the nature and level of fear of COVID-19 among OHP’s in South Africa.

## Research methods and design

### Study design

An online cross-sectional survey was undertaken using Google Forms, which were distributed to OHPs in South Africa. This study design strategy was most appropriate during the pandemic to reach participants online and not physically. Additionally, questionnaires are most suitable in assessing self-reported attitudes and perceptions in an unbiased manner.

### Setting

Participants were OHPs in South Africa, who were reached through online platforms.

### Study population

Oral health professionals, inclusive of dental specialists, dentists, dental therapists and oral hygienists, were included in the study irrespective of the employment sector. Current registration with the regulator, the Health Professional Council of South Africa (HPCSA) was a critical inclusion criterion to enable stratification.

### Sampling and sample size

According to the 2021 registration data from the HPCSA, a total of 8056 OHPs were licensed to practice in South Africa. Considering this universe, the sample size was calculated as a minimum of 358 under the following assumptions: (1) 5% margin of error, (2) level of precision of 95% and (3) 58.3% of dentists presented with fear and anxiety to COVID-19 (Suryakumari et al. 2022). To determine the minimum sample size for each cadre, the study population (*n* = 358) was further stratified according to the professional grouping. Consequently, the estimated numbers were 293 for dentists and dental specialists, dental therapists (35) and oral hygienists (63). These figures correspond to 4% of 6059, 740 and 1257 of the registered OHPs for each grouping, with an additional 20% to counteract non-response rate. The online survey remained open until the required sample size was reached for each professional grouping.

### Measurement of variables

The widely applied and validated fear scale of COVID-19 (FCV-19S) was used in the study to assess the level of fear among oral health workers (Alimoradi et al. 2022). For this study, four items of the fear scale were assessed on a five-point Likert scale. The scores ranged between 1 and 5, representing the absence of fear to extreme fear. The levels of fear were recorded as follows: 1 = Not at All (NAA), 2 = Just a Little (JAL), 3 = Indifferent (IND), 4 = Pretty Much (PM) and 5 = Very Much (VM). The total scores ranged between 4 and 20, with the higher score, signifying the higher levels of fear of COVID-19. Demographic information collected included age, sex, type of practice and years of practice. The participants also indicated whether they trained as dentists, dental specialists, dental therapists or oral hygienists. The history of COVID-19 testing and vaccination was also ascertained.

### Data collection

The link to the form was sent to all the dental associations in the country, which used their databases to source emails and or cell phone numbers. These details were used to circulate the link to prospective participants. Informed consent was sought from participants before they could complete the questionnaire. The questionnaire took approximately 5 min to complete, and online survey remained open until the required sample size was reached for each professional grouping. Several reminders were sent throughout the duration of the study. Data collection started in February 2022 and continued until November 2022.

### Data analysis

Data were analysed using SPSS ver. 28 software. Appropriate descriptive analyses were computed for each variable under the study. Ratio and interval variables were subjected to measures of central tendency and dispersion, together with tests of normality. Nominal and ordinal variables were subjected to measures of frequency or proportion. The median value for the composite fear score was used to indicate the presence or absence of fear. Scores greater than the median (> 15) were categorised as the presence of fear and less than or equal to the median as an absence of fear of COVID-19. Chi-square tests and analysis of variance (ANOVA) were performed (Table 2, Table 3). Variables that were deemed significant (α ≤ 10%) were fitted in the multivariate logistic regression model.

### Ethical considerations

Ethics approval to conduct this study was granted by Sefako Makgatho Health Sciences University Research and Ethics Committee (SMUREC/D/113/2021:PG). Participants consented to take part in the survey, and anonymity and confidentiality were ensured throughout the conduct of the study. The results are aggregated. The findings can therefore not be linked directly to individual participants.

## Results

Table 1 depicts the sociodemographic characteristics of 462 OHPs. A total of 326 (70.6%) females, 267 (57.8%) dentists and 38 (8.2%) dental specialists participated in the study. The majority of OHPs, 265 (57.4%), were in private practice. The average age and clinical experience of the OHPs were 39.95 (11.61) and 14.42 (11.28) years, respectively. Enquiries about COVID-19 history revealed that 191 (41.3%) of participants tested positive for COVID-19 and 373 (81.6%) were vaccinated. According to Table 2, a significant majority of OHPs 293 (63.5%) were not afraid of contracting COVID-19. However, 139 (51.7%) feared infecting their friends and families; 258 (55.8%) feared hospitalisation because of COVID-19, and 239 (51.8%) were apprehensive of dying because of COVID-19. Approximately half of the OHPs, 212 (45.6%) indicated having severe fear of COVID-19. The overall logistic regression model was statistically significant when compared to the null model (χ^2^ = 52.75, *p* < 0.000). The variables, age (*p* = 0.03), clinical experience (0.036), employment sector (0.018), professional group (0.021), positive COVID-19 test (*p* = 0.003) and COVID-19 vaccination (*p* < 0.000) were significant predictors of the COVID-19 fear (Table 3). Specifically, the level of corona phobia rose with increasing age; the odds ratios were 2.13 and 2.34 for age groups 31–40 and over 40 years, respectively. Dental therapists were two or three times more likely to be fearful and anxious about COVID-19 compared to oral hygienists (OR = 2.73). On the contrary, OHPs with positive COVID-19 tests were less apprehensive (OR = 0.58), while the vaccinated cohort showed comparatively high levels of phobia (OR = 3.23).

**TABLE 1:** Demographic characteristics of Oral Health Professional.

Variables	Category	*n*	%	Mean	SD	Range
**Age (years)**	-	-	-	39.91	11.69	19–73
	≤ 30	120	26.0	-	-	-
	31 – 40	147	31.8	-	-	-
	> 40	195	42.2	-	-	-
**Gender**						
	Female	326	70.6	-	-	-
	Male	136	29.4	-	-	-
**Profession**						
	Dental specialist	38	8.2	-	-	-
	Dentist	267	57.8	-	-	-
	Dental therapist	54	11.7	-	-	-
	Oral hygienist	103	22.2	-	-	-
**Sector**						
	Private practice	265	57.4	-	-	-
	Public service	129	27.9	-	-	-
	Academic	68	14.7	-	-	-
**Experience**	-	-	-	14.42	11.28	1–50
	≤ 10	220	47.6	-	-	-
	11 – 20	114	24.7	-	-	-
	> 20	128	27.7	-	-	-

SD, standard deviation.

**TABLE 2:** Responses to fear and anxiety questions.

Variables	NAA		JAL		IND		PM		VM	
	*n*	%	*n*	%	*n*	%	*n*	%	*n*	%
Q1. I am afraid of getting infected by Covid-19	109	23.6	131	28.4	53	11.5	101	21.9	68	14.7
Q2. I am fearful that I could carry infection from my workplace back to my family	74	16.0	115	24.9	34	7.4	118	25.5	121	26.2
Q3. I am afraid of getting hospitalised because of Covid-19	84	18.2	87	18.8	33	7.1	100	21.6	158	34.2
Q4. I am afraid of dying due to Covid-19	130	28.1	55	11.9	38	8.2	77	16.7	162	35.1

Note: Overall (composite) fear score: Mean = 12.50; SD = 5.26; Me = 13.00.

NAA, not at all; JAL, just a little; IND, indifferent; PM, pretty much; VM, very much.

**TABLE 3:** Logistic regression analysis.

Variables	OR	95% CI	*p*-value
**Age group (years)**			
≤ 30	1.00	-	-
31 – 40	2.13	1.18–3.85	0.013*
> 40	2.34	1.06–5.19	0.036*
**Gender**			
Female	1.00	-	-
Male	0.71	0.45–1.13	0.150
**Profession**			
Oral hygienist	1.00	-	-
Dental therapist	2.72	1.33–5.75	0.006*
Dentist	1.33	0.78–2.67	0.290
Dental specialist	1.36	0.59–3.13	0.470
**Experience (years)**			
≤ 10	1.00	-	-
11–20	1.04	0.57–1.88	0.910
> 20	0.44	0.2–0.96	0.039*
**Employment sector**			
Private	1.00	-	-
Public	1.97	1.22–3.17	0.005*
University	1.15	0.64–2.06	0.640
**COVID-19 test**			
Negative	1.00	-	-
Positive	0.58	0.37–0.90	0.016*
**COVID-19 vaccination**			
No	1.00	-	-
Yes	3.23	1.87–5.59	< 0.001*

* , Statistically significant.

## Discussion

Overall, 45.6% of South African OHPs had a severe fear of COVID-19 (Table 2). This figure is low compared to 57.8% (Collin et al. 2021), 67% (Prince et al. 2021), 78% (Bírant & Gümüştaş 2022), 88% (Kamran, Saba & Azam et al. 2021) and 90% (Rauf et al. 2021), but higher than 42.69% (Suryakumari et al. 2022). The authors attribute the relatively moderately subdued levels of fear in South Africa to be indicative of the success in dealing with the COVID-19 pandemic. Compared to its counterparts in the Southern African Development Community (SADC), South Africa devoted sufficient resources in managing the pandemic. The country, employed a world-class surveillance system, implemented impeccable tracking and tracing processes and established several centres for the clinical management of the infected patients (Moonasar et al. 2022). South Africa was the first African country in the region to implement a large-scale vaccination programme for its citizens (Dzinamarira et al. 2021). Therefore, the variations in severity of fear among OHPs in several countries could reflect the differences in the country’s attitude, resolve and attempts in managing the COVID-19 pandemic. Overall, countries that demonstrated relative success in dealing with the pandemic succeeded also in alleviating national fear, anxiety and panic. The early phases of COVID-19 in South Africa were characterised by generalised fear, anxiety and hopelessness in the face of uncertainty. However, as knowledge about the virus accumulated and effective treatments became available, the levels of fear lessened. This South African study was conducted from February 2022 until November 2022. During this period, the country went through the highest peak (third wave) and decline (fourth wave) and recorded a total of 101 219 COVID-19 deaths and about 4 million cases (NICD 2022). A nationwide vaccination programme was implemented; however, misinformation remained rampant giving rise to vaccine hesitancy. Despite these challenges, the fear of COVID-19 moderated slightly, especially among health professionals including OHPs in South Africa (Dubé et al. 2013).

About two-thirds (63.5%) of OHPs in South Africa were afraid of contracting the virus, while 51.7% were afraid of infecting family and friends (Table 2). Comparatively, these South African levels are lower than OHPs from Turkey (78.0% and 88.3%) (Bírant & Gümüştaş 2022), Pakistan (75% and 92%) (Kamran et al. 2021) and India Jabalpur (67% and 84.04%) who reported elevated levels of COVID-19. The authors hypothesise that the following factors explain the comparatively low coronaphobia among OHPs in South Africa: (1) the national COVID-19 response was well established and matured (Moonasar et al. 2021); (2) the pandemic was slowing down, evidenced by the flattening of the curve (Moonasar et al. 2021); (3) natural immunity and vaccination levels were adequate to provide modest herd immunity (Zar et al. 2022); (4) the dominant strain of the fourth wave, the Omicron, while highly transmissible was less virulent (Al Hasan et al. 2022) and (5) the majority of practices had closed down or offered limited dental services, thus reducing the risks of infections (Koutras et al. 2020). Practices that continued to offer dental services enforced stringent infection control and prevention measures, including the use of PPEs. Therefore, as the pandemic matured and the national response succeeded, levels of fear of COVID-19 lessened among the OHPs and population at large.

Our findings indicate that males had comparatively less fear of COVID-19 than females (Table 3). This result is similar to the study conducted among healthcare professional (Osagiator Ariyo et al. 2021). Female university students were less afraid of COVID-19 (Morales-Rodríguez 2021), while among hospital staff showed no gender differences in corona phobia (Ashoor et al. 2021). The authors postulate that gender roles might exacerbate fear in females. Males are socialised to act strong while females are encouraged to express emotions, including fear (Nino et al. 2021). Additionally, biological factors such as hormonal and reproductive changes may mediate differences in emotional responses to stimuli, including fear (Pigott 1999). A comprehensive meta-analysis by Metin et al. (2022) validated the finding that the COVID-19 pandemic had worse psychological effects on females compared to males.

The fear of COVID-19 among OHP’s increased with increasing age. The odds ratios of 2.13 and 2.34 were observed for older age groups compared to those younger than 30 years of age (Table 3). This variation can be ascribed to perceived vulnerability and real risk of COVID-19 infection in these age groups. The severity and fatality of COVID-19 have been found to be higher in the elderly with compromised immunity and pre-existing physical conditions (Alimoradi et al. 2022; Jain & Jha 2020). It would therefore be expected that older OHP’s would be more fearful given their susceptibility to the infection and clinical outcomes (Barnett et al. 2020; Barranco & Ventura 2020).

An overall significant difference in COVID-19 fear scores according to clinical experience was reported in this study (Table 3). The levels of fear decreased with increasing clinical experience, OR = 1.04 (11–20 years) and 0.44 (> 20 years), compared to the reference group (≤ 10 years). This finding is contrary to studies by Van de Venter et al. (2021), Elshami et al. (2021) and Hu et al. (2020) who found similarities in fear levels across levels of clinical experience. A plausible account for this result is that the experienced clinicians are more discerning, have critical expertise, are more prepared for uncertainty and are able to traverse ‘unknown’ terrain. Therefore, the number of years in clinical practice prepared the OHPs to adapt and pivot easily even during the pandemic.

There was a significant correlation between coronavirus-related fear and employment sector. Oral health professionals in private practice were less fearful compared to those employed in other sectors. These practitioners who seek out their living in private surgeries do not have the opportunity nor the inclination to be paralysed by fear and abscond from their work. Their livelihood depends in keeping their fingers wet and hands in patient’s mouths whatever the costs. The motivation for OHP’s in private practice to overcome fear is far greater and necessary that clinicians in any other sector.

A positive association was found between vaccination and COVID-19 fear (OR = 3.23) (Table 3). This relationship is well explained in several publications (Awijen, Ben Zaied & Nguyen 2022; Bono et al. 2021; Willis et al. 2021). According to the health belief model (Rosenstock 1974) and protection motivation theories (Rogers 1975), fear is an independent predictor of health-related behaviours such as vaccination, wearing of masks, etc. Therefore, the perception of risk and vulnerability for self, family and friends can act as a significant stimulus to vaccinate against the virus. Given the nature of the study design, this concurrent, cross-sectional study cannot establish a temporal relationship between these variables. Therefore, this association cannot conclusively ascertain whether fear of COVID-19 predicts vaccination or whether vaccination status impact on fear of COVID-19.

Oral health professionals who tested positive for corona virus exhibited less fear than those with negative results (OR = 0.58) (Table 3). This result suggests that contracting COVID-19 does not increase the level of fear experienced. Longitudinal studies have shown that in the short term (14 days of testing positive), the levels of distress are heightened. However, in the post-symptomatic phase, the levels of fear subside significantly (Oyem, Wang & Viguera 2021). The acute phase of COVID-19 infection increased fear attributed to the advent of symptoms, clinical sequelae and unknown prognosis (Taquet, Holmes & Harrison 2021a; Taquet et al. 2021b). Overall, the findings of the present study are consistent with cross-sectional studies linking COVID-19 symptoms to measures of mental health (Hyland et al. 2021; Wathelet et al. 2020).

### Limitations

This cross-sectional study design is prone to information and selection bias namely recall bias and prevalence-incidence. It could not be established in this study if biases contributed to the under or overestimation of the effect size. Over the course of the disease, distress dissipated and fear levels dropped as OHPs began to have a handle on their disease state. Despite these limitations, the study was adequately powered and used a validated tool, which limits threats to validity.

## Conclusion

Our findings show that South African OHPs had low levels of COVID-19 fear. On the contrary, females, older participants and vaccinated OHPs had the greatest fear of COVID-19.
